# Quality and Safety of Vaccines Manufacturing: An Online Survey on Attitudes and Perceptions of Italian Internet Users

**DOI:** 10.3390/vaccines9091015

**Published:** 2021-09-13

**Authors:** Angela Bechini, Beatrice Zanella, Benedetta Bonito, Sonia Paoli, Giulia Di Pisa, Andrea Moscadelli, Leonardo Ancillotti, Paolo Bonanni, Sara Boccalini

**Affiliations:** 1 Department of Health Sciences, University of Florence, 50134 Florence, Italy; angela.bechini@unifi.it (A.B.); beatrice.zanella@unifi.it (B.Z.); benedetta.bonito@unifi.it (B.B.); leonardo.ancillotti@stud.unifi.it (L.A.); paolo.bonanni@unifi.it (P.B.); 2 Medical Specialization School of Hygiene and Preventive Medicine, University of Florence, 50134 Florence, Italy; sonia.paoli@unifi.it (S.P.); giulia.dipisa@unifi.it (G.D.P.); andrea.moscadelli@unifi.it (A.M.)

**Keywords:** hesitancy, vaccination, Italy, perceptions, healthcare workers, questionnaire, parents

## Abstract

*Background:* Vaccination is a worldwide public health practice that requires high uptake levels in order to effectively reduce the incidence of vaccine-preventable diseases. The manufacturing of vaccines is a complex process, and little is known about people’s feelings and opinions on that. Our study aimed at investigating perceptions and attitudes of the general population towards the vaccine production process before the availability of COVID-19 vaccines. *Methods:* We designed a 15-question online survey in the Italian language which was spread via Facebook and an Italian website "Vaccinarsintoscana" between January and May 2020. We performed a descriptive analysis and applied statistical tests to assess differences in the given answers according to participants’ sociodemographic characteristics. *Results:* The collected responses (135 participants) about the perceptions on vaccine production process were largely positive: not being concerned about the vaccine production (70.3%); believing the vials did not contain harmful substances (75.6%) and considering the precautionary withdrawal of some batches as highly effective (83.7%). In contrast, a less positive perception was found for the question about the conflict of interest between manufacturing companies and the control systems (48.9%). Moreover, people’s perceptions towards the vaccine components (i.e., microorganism, adjuvants and opinion on batches withdrawal) also showed a good level of confidence and trust. *Conclusions:* Our study highlighted a generally positive attitude towards the vaccine production process and showed people’s confidence in the control systems, safety and high standards of quality of the vaccine production process.

## 1. Introduction

The introduction of vaccination as a global clinical practice is one of the most important public health achievements that has drastically reduced the morbidity and mortality rates of many infectious diseases. Recently, the World Health Organization (WHO) has estimated that vaccinations prevent up to 3 million deaths each year worldwide [1]. Vaccination is based on the administration of agent-specific yet relatively harmless antigenic components that in vaccinated subjects may induce protective immunity against the corresponding infectious agent. Vaccines are generally very safe, and serious adverse reactions are uncommon. Routine immunization programs protect most children from a number of infectious diseases that previously caused millions of deaths each year (i.e., poliomyelitis and smallpox) [1,2]. The strength and, at the same time, the weak point of vaccines is that they must receive high uptake levels in order to successfully reduce the incidence of vaccine-preventable diseases; otherwise, herd immunity cannot be reached [3]. Nevertheless, during the last few decades, as a consequence of the huge reduction of infectious diseases that followed the vaccination programs all over the world, a part of the general population became hesitant towards vaccination in Italy, too [4].

In the context of vaccination prevention, general population attention and fears are focused on efficacy and safety issues concerning the development of vaccines through clinical trials and adverse events following immunization. Less attention addressed safety and quality issues during vaccine production. In a recent study in Italy, the knowledge on this topic was investigated [5]. As a matter of fact, the manufacturing process of vaccines is a complex process people are not completely aware of. Vaccine companies must comply with Good Manufacturing Practices (GMP). As a consequence, many special quality controls (QC) and quality assurance (QA) systems are required in all steps of the process. Therefore, the production of a vaccine may last from 6 to 36 months [6,7]. QC assessments are based on the general guidelines for biologicals set up by the WHO and the National Control Authorities (NRAs). To underline the importance of quality controls, time dedicated to QC is 70% of the whole production timeline [8]. Tests have been developed and validated to demonstrate the purity of the cell substrate used for each production cycle, the quality of the microorganism harvest, the adequacy of all the processes and the conformity to stringent specifications for purity, safety and potency of the final bulk vaccine filled in final containers [9]. Despite the quality and safety of vaccines manufacturing that may influence trust and confidence towards vaccinations, this issue has not yet been extensively investigated. Health care professionals and people working in the vaccine companies are surely aware of these practices, but little is known about people’s feelings and opinions on the actual manufacturing process.

The main objective of this study, performed before the availability of COVID-19 vaccines, was to investigate people’s perceptions and attitudes towards the vaccine production process.

## 2. Materials and Methods

### 2.1. Study Design and Questionnaire Dissemination

This cross-sectional study was carried out through an online questionnaire in Italian developed on Google Modules application. Firstly, a dedicated focus group was established. It was composed of two university professors, experts in vaccines, university students (nursing and humanistic studies), residents of the Medical Specialization School of Hygiene and Preventive Medicine at the University of Florence, a scientific journalist and a member of a vaccine-manufacturing pharmaceutical company. After the focus group discussion, the main topics about attitudes and opinions on the vaccine production process and vaccine components were identified. Subsequently, a pilot questionnaire was tested on about 20 subjects. Participants’ positive and negative feedbacks were used to modify and improve the final version of the questionnaire. The survey was spread through Facebook and an Italian website dedicated to vaccination (Vaccinarsintoscana: https://www.vaccinarsintoscana.org/, accessed on 10 September 2021) during the period January 2020–May 2020. Participants could share the link to the questionnaire with their contacts through social platforms or instant messaging applications.

The only inclusion criterion was to provide consent to participate in the study. The questionnaire was addressed to subjects aged ≥18 years, internet users and social media users.

The survey consisted of four sections:

1. Sociodemographic information: age (analyzed by age group: 20–29 years, 30–39 years, 40–49 years, >49 years), sex, work or education in the healthcare setting (work/education: HC or not HC) and having offspring (minors/no minors);
2. Perceptions and attitudes towards vaccine production: this section included four 5-point Likert scale statements ranging from “totally disagree” to “totally agree” and one open question;
3. Perceptions and attitudes towards vaccine components: this section included yes/no and multiple-choice questions;
4. Information about vaccines and their production: included one yes/no and one multiple-choice question.
5. Questionnaire is available as a Supplementary Materials File S1: Informed Consent, Questionnaire and Tables.

### 2.2. Statistical Analysis

Answers were automatically collected into a database and subsequently analyzed using IBM SPSS Statistics 26.

The enrolled population was divided into different groups according to sociodemographic information: age, sex, type of work or education (healthcare and not healthcare setting) and offspring (minors and non-minors). A descriptive analysis was performed to characterize the study population and to evaluate the frequencies and the percentages of the collected answers according to the sociodemographic groups. A chi-square test was applied to assess significant differences among the answers, according to the different sociodemographic characteristics of the participants. A *p*-value ≤ 0.05 was considered statistically significant.

Answers for 5-points Likert scale statements were encoded, assigning a score ranging from 1 to 5 as follows:

- For affirmative statements, “1” corresponded to “totally disagree and “5” corresponded to “totally agree”;
- For negative statements, “1” corresponded to “totally agree” and “5” corresponded to “totally disagree.

To assess the normal distribution of Likert score, we applied the Kolmogorov–Smirnov test, which confirmed a non-normal distribution (*p*-value < 0.05). We applied the non-parametric Kruskal–Wallis test to assess significant differences in the distribution of Likert scores for each statement related to the different sociodemographic characteristics. A *p*-value ≤ 0.05 was considered statistically significant.

Moreover, we calculated the mean of the Likert scores assigned to each participant, and then we applied the Spearman′s rank correlation coefficient to assess a likely correlation between the level of agreement toward the vaccine production process and the sociodemographic characteristics of our study population.

Lastly, the Cronbach′s alpha coefficient of reliability was applied to measure the internal consistency of the 5-point Likert scale statements [10].

### 2.3. Multivariate Logistic Regression Models

Two different multivariate logistic regression models were applied to assess whether sociodemographic characteristics (age, sex, professional profile and having offspring) would predict attitudes or perceptions towards the vaccine production process and the vaccine components. The first logistic regression model was applied on the 5-points Likert scale statements (second section of questionnaire) to probe the association between sociodemographic characteristics and attitudes toward vaccine production process. The collected responses to each question were dichotomized into positive (scores 5, 4) versus non-positive (scores 3, 2, 1) [10]. The second logistic regression model was performed on yes/no and multiple-choice questions to assess an association between sex, age, professional profiles in health care settings and having offspring (independent variables) and the attitudes toward vaccine components. Questions with yes/no answers were implemented in a binary logistic regression model, and the questions with multiple answers were all encoded as “positive/non-positive” attitudes in the same regression model. The odds ratio was adjusted for all the independent variables, resulting in an adjusted odds ratio (AOR). The Hosmer–Lemeshow goodness-of-fit statistics (H–L test) were used to assess whether the model adequately described the data.

## 3. Results

### 3.1. Study Population

A total of 136 questionnaires were collected during the period January 2020–May 2020. Only one subject did not give consent to participate; thus, we included 135 surveys in our analysis. Participants were mostly females (65.2%; 88/135) and mainly <39 years old (74.3%). The youngest respondent was 21 years old, whereas the oldest was 69 years old, and the mean age was 35.4 years. Most subjects did not have an education or did not work in the healthcare setting (65.2%; 88/135) and did not have minor offspring (71.9%; 97/135) (Table 1).

### 3.2. Perceptions and Attitudes toward the Vaccine Production Process

**Figure 1:**
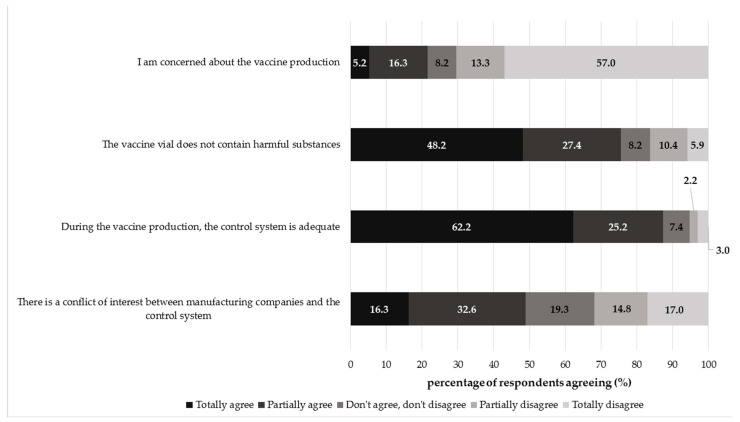
Overall percentage distribution of respondents agreeing regarding the vaccine production process. Cronbach′s alpha coefficient = 0.411.

The collected perceptions towards the vaccine production process were largely positive, except for the conflict of interest issue (Figure 1). Most participants disagreed with the statement “I am concerned about the vaccine production process” (totally disagree: 57.04%, 77/135; partially disagree: 13.33%, 18/135). Moreover, a consistent part of respondents agreed that the vaccine vial did not contain harmful substances (totally agree: 48.15%, 65/135; partially agree: 27.41%, 37/135) and that during the vaccine manufacturing, the control system was adequate (totally agree: 62.22%, 84/135; partially agree: 25.19%, 34/135). On the other hand, a generally negative perception was found for the last statement; indeed, about 49% of the respondents agreed that there was a conflict of interest between manufacturing companies and the control systems (totally agree: 16.30%, 22/135; partially agree: 32.59, 44/135) and 19% of participants gave a neutral answer (do not agree/do not disagree: 19.26%, 26/135). Less than 32% of subjects disagreed that the conflict of interest existed (partially disagree: 14.81%, 20/135; totally disagree: 17.04% 23/135).

Table 2 summarizes the respondents’ perceptions about the vaccine production process stratified by sociodemographic characteristics. There was a general disagreement to be concerned about the vaccine production process, with comparable results in the given answers in the different groups. No statistically significant differences were found. Males, subjects who had an education or worked in the healthcare setting and those who did not have minor offspring were more confident that the vaccine vial did not contain harmful substances (totally agree: 59.57%, 59.57%, 50.22%, respectively), and that the control system was adequate during the vaccine production (totally agree: 72.34%, 34/47; 72.34%, 57.89%, respectively) compared to females and participants who did not have an education or work in the healthcare setting and those who had minors. No statistically significant differences were found among these groups. Concerning the conflict of interest between pharmaceutical companies and the control systems, comparable percentages in the given answers were found among males and females, with a general trend of a non-positive attitude. The same outcome is shown comparing answers given by subjects with/without minors, whereas, in this case, the differences found were statistically significant (chi-square test: *p*-value = 0.04;) Kruskal–Wallis: *p*-value = 0.003). On the other hand, a higher number of participants who had an education or worked in the healthcare setting totally disagree with the statement “There is a conflict of interest between manufacturing companies and the control system” (31.91%, 15/47), compared to subjects who did not have an education or work in the healthcare setting (9.09%, 8/88); this difference was statistically significant (chi-square test: *p*-value < 0.001; Kruskal–Wallis: *p*-value < 0.001). The Spearman′s rank correlation coefficient showed a negative correlation, even if weak, between the agreement toward the vaccine production process and not having had an education or worked in the healthcare setting (−0.227, *p*-value = 0.008).

The analysis of the attitudes toward the vaccine production process stratified by age group highlighted similar results in the different groups for each statement, and no statistically significant differences were found (Table S1 in Supplementary Materials File S1: Informed Consent, Questionnaire and Tables). Nevertheless, in some cases, we retrieved more positive attitudes among young subjects, such as for the statements “I am concerned about the vaccine production” (totally disagree: 66.67% in 20–29 years old group compared to 33.33% in >49 years old group) or “There is a conflict of interest between manufacturing companies and the control system” (totally disagree: 23.53% of subjects aged 30–39 years old compared to 11.11% of participant aged 40–49 years old). In other circumstances, older subjects seemed to have more positive perceptions, such as for the sentences “The vial does not contain harmful substances” (totally agree: 55.56% of subjects aged >49 years old compared to 45.83% of participants aged 20–29 years) or “During the vaccine production, the control system is adequate” (totally agree: 77.78% of subjects aged 40–49 years old compared to 60.42% of participants aged 20–29 years) (Table S1 in Supplementary Materials File S1: Informed Consent, Questionnaire and Tables).

The last question (open short answer) of Section 2 of the survey was about the main fears toward vaccination. We collected 75 answers: 34.6% of the respondents confirmed no fear toward vaccines, while 65.4% reported at least one concern. The most-reported worries were adverse events following immunization (23%) and the presence of contaminants or harmful substances (18%), followed by a conflict of interest (13%). Other answers included lack of controls (12%), lack of efficacy (5%), human errors (3.3%), concern about unsuitable pre-vaccination anamnesis and the need to improve knowledge about vaccine production (3.3%), and fears of bad storage of the vials (1.7%).

### 3.3. Perceptions and Attitudes towards Vaccine Components

Table 3 summarizes the answers collected in the third section of the questionnaire. Overall, participants’ perceptions toward vaccines and vaccines components were positive: about 82% of them (111/135) thought that the microorganisms from which the vaccine is derived were adequately treated to make the vaccine harmless and unable to cause the disease, and about 84% (113/135) considered a precautionary withdrawal of vaccine batches as a high and effective control system. Meanwhile, lower confidence was found toward the adjuvants: even if more than half of participants (57.8%, 78/135) thought that adjuvants were not harmful, about 13% of subjects thought the contrary, and a consistent part of participants (29%, 39/135) answered that they did not know what an adjuvant was. The main concerns about adjuvants revealed the respondents’ opinion that adjuvants were not controlled (32%, 7/22) or that their amount was too high (36%, 8/22). Only a few subjects affirmed that adjuvants were harmful compounds (14%, 3/22) or had concerns about the possibility of an allergic reaction or of an adverse event following immunization (9%, 2/22). Percentages of the collected answers were comparable between males and females and between subjects who had children and who did not; indeed, no statistically significant differences were found, whereas differences were found comparing the answers given by participants who had an education or worked in the healthcare setting and by subjects who did not. In fact, most participants who had an education or worked in the healthcare setting thought that vaccines derived by microorganisms are not harmful and do not cause the disease compared to subjects who did not (95.7% and 75%, respectively; *p*-value < 0.001). Almost all the subjects who had an education or worked in the healthcare setting (about 96%) thought that the precautionary withdrawal of vaccine batches was a strong and effective control system compared to about 77% of not HC subjects (*p*-value < 0.001). Subjects who had an education or worked in the healthcare setting seemed to have more positive attitudes toward the adjuvants; moreover, few participants belonging to this group did not know what an adjuvant was, compared to not HC subjects (2.13% and 43.2%, respectively, *p*-value = 0.01).

Lastly, the analysis of the attitudes and perception toward vaccine and vaccine components according to the age highlighted comparable percentages for each statement comparing the age groups. Thus, positive attitudes seemed to be equally distributed in the age groups, and no statistically significant differences were found in the given answers (Table S2 in Supplementary Materials File S1: Informed Consent, Questionnaire and Tables).

### 3.4. Information about Vaccines and Their Production

Most participants answered they were interested in attending meetings about vaccines and their production (60%; 81/135), while about 12% of them were not interested and about 28% answered that this would depend on who will organize them. The last question (with multiple-choice answers) retrieved 119 answers and 295 different combinations of choices about who should organize these events. Subjects mainly thought the Minister of Health (31%, 91/295) or educational institutes such as schools or universities (23%, 69/295) should organize them, followed by the Order of Physicians and local organisms as regions or municipalities (17%, 50/295 and 16%, 48/295, respectively), then pharmaceutical companies (11%, 31/295). Other choices were local health units, the Italian Medicines Agency (AIFA) or external bodies (2%, 6/295).

### 3.5. Multivariate Logistic Regression Models

We developed two different multivariate logistic regression models to assess the likelihood that sociodemographic characteristics could predict people’s attitudes towards the vaccine production process (first model) and towards the vaccine and its components (second model). For Section 2 of the questionnaire (5-point Likert Scale), only the statement about the conflict of interest resulted in having one of the independent variables being almost statistically significant. In particular, working or having an education in the healthcare setting was a predictor for disagreement toward the possibility of conflict of interest between pharmaceutical companies and the control system (AOR = 2.43; CI: 1.08–5.48; *p*-value = 0.03). In addition, having minor offspring could be considered a predictor factor. Indeed, we found that subjects who had minors were more inclined to believe that a conflict of interest may exist (AOR = 0.25; CI: 0.08–0.74; *p*-value = 0.01) (Table 4).

Table 5 shows the sociodemographic predictors for participants’ attitudes toward vaccine and vaccines components. We found that working or having an education in the healthcare setting was a predictor for considering the vaccines derived by microorganisms as not harmful (AOR = 9.80; CI: 2.05–46.92 *p*-value = 0.004), the presence of adjuvants as not dangerous (AOR = 6.84; CI: 2.63–17.82; *p*-value < 0.001) and the precautionary withdrawal of some batches of vaccines as highly effective (AOR = 8.38; CI: 1.79–39.31; *p*-value = 0.007). Lastly, sex can also be considered as a predictor factor, in particular, males were more confident to consider that microorganisms from which the vaccine is derived is adequately treated and unable to cause the disease (AOR = 3.61; CI: 1.10–11.84; *p*-value = 0.03). The Hosmer and Lemeshow test confirmed the satisfactory GOF (goodness-of-fit) for both the models (Table 4 and Table 5).

## 4. Discussion

In this study, we have described people’s attitudes and perceptions towards the quality and safety of the vaccine production process in a convenient sample during the first phase of the COVID-19 pandemic in Italy.

Our online survey showed a generally positive attitude and good confidence regarding vaccine manufacturing: i.e., 70.3% of respondents were totally and partially concerned about the vaccine production, 75.6% totally and partially agreed with the absence of harmful substances in the vaccine vial and 87.4% trusted control authorities during the production process. These positive attitudes resulted independently from sex, work/education in HC and having minor offspring. However, almost half of the participants totally or partially agreed that there was a conflict of interest between manufacturing companies and control systems. Importantly, we found a statistically significant difference in conflict of interest between people having or not having minor offspring (*p* < 0.05) and people working/educated in HC and not HC (*p* < 0.001). Actually, we found that subjects who had minors were more inclined to believe that a conflict of interest may exist (totally agree 28.95% vs. 11.34%); similarly, subjects not working/educated in HC agreed more with the conflict of interest (totally agree 22.73% vs. 4.26%). Thus, it is remarkable how having minor offspring and, more importantly, having a healthcare background, may impact the positive perceptions and attitudes towards the vaccine production process.

Moreover, the questionnaire section dedicated to the perceptions about the vaccine components showed good confidence levels: i.e., 82.2% of respondents believed that the microorganisms from which the vaccine is derived are adequately treated and harmless, and almost 60% considered the amount of adjuvants in vaccines not dangerous. Finally, about 84% of respondents considered batches withdrawal as a highly effective control measure. Particularly, the statistically significant differences concerning the vaccine components among the group of people working/educated in the healthcare setting and those who were not are relevant: the HC respondents resulted more confidently about the vaccine components. This is a very relevant finding considering that healthcare workers (HCWs) are often referred to as the most trusted source of vaccine-related information [11,12]; in our previous study, good levels of knowledge about vaccine manufacturing were found [5]. On the other hand, other factors may negatively influence healthcare attitudes toward vaccines. For example, a significant prevalence of vaccine-hesitant French nurses was found among those with high vaccine risk perception or low trust in governmental bodies, such as the Ministry of Health [13].

The other group of participants who showed a less positive attitude toward vaccine safety were parents. The reasons behind this concern are particularly unknown adverse reactions that might develop long after vaccination. These fears often arise around the novelty and type of vaccines and claim that they have not been tested long enough [9]. In other cases, some cultural aspects, such as religious or moral barriers to vaccination, have also been described, including concerns about the prior use of fetal tissues in the manufacturing of vaccines [14]. In our study, parents also show concern about the possible conflict of interest between manufacturing companies and control systems. Indeed, besides the safety issue [15], our results confirm that one of the major concerns arising among individuals who have doubts about vaccinations is that there may be commercial interests by those who produce vaccines. These findings seem to be in line with the idea that vaccines are introduced to serve the economic and/or political interests of pharmaceutical companies, western countries and governments [14,16]. Particularly, our results showed a certain degree of concern about the conflict of interest between the manufacturing companies and the control systems. As a matter of fact, the control procedures are carried out independently by the manufacturing companies and also by certified laboratories and regulation authorities. In order to overcome the conflict of interest issue, proper information is recommended to be provided to the general population. Considering that the respondents to our survey were new media users, a digital promotion should be specifically designed using these new tools. As far as the vaccine components, our results have shown a certain degree of concerns about adjuvants, while 28.89% of the participants did not know what an adjuvant was. This disbelief and knowledge gap may also reduce vaccination compliance, as reported in the literature [17,18]. Thus, among the most common fears, there are that some components (adjuvants such as aluminum, preservatives such as mercury, inactivating agents such as formaldehyde, manufacturing residuals such as human or animal DNA fragments and simply the sheer number of vaccines) might be overwhelming, weakening or perturbing the immune system. Aluminum is used as an adjuvant to boost the immune response to the vaccine antigens and is used in a variety of vaccines, including hepatitis A and B, H. influenzae type b and pneumococcal vaccines [19]. The safety of adjuvants has been evident in clinical trials for many decades [20,21,22,23]. In order to further mitigate the worries about adjuvants, specific communication activities should address that the quality control processes are also carried out on each individual component of vaccines, such as adjuvants. Therefore, these elements underwent rigorous quality control procedures as well during the production phase.

Women and subjects with a healthcare background were the two groups who mainly represented our sample composition (about 65% and 35%, respectively). A possible explanation for this takes into account the way the survey was spread: the regional website VaccinarSinToscana is indeed mainly referred by young women. Moreover, the healthcare community (such as pediatricians or general practitioners) actively looks for updated information about vaccines and vaccinations on this platform [24].

The small number of participants and the way the questionnaire was spread (via Facebook and the internet) may limit the quality of the study. The choice to spread the questionnaire via social media may have limited the representativeness of the sample, excluding subjects that do not use new media or those who do not have internet access, as well as all people not interested in social networks. A third possible sampling bias may be due to the polarization of our study population: it has been reported that online users tend to select information closed to their beliefs, ignoring information in contrast with their opinion [25]. Thus, the good confidence level toward the vaccine production process we found may have been affected by the biases discussed above. Moreover, this survey has the availability during the first phase of the COVID-19 pandemic in Italy, at a time in which the public attention was directed mainly towards the health emergency, rather than to other topics of public health interest, such as vaccines. In that period, no COVID-19 vaccines were available. As a matter of fact, the questionnaire adhesion was sub-optimal, and our sample size was not large due to the earlier withdrawal of the questionnaire from the web. At this point, we cannot increase the number of respondents to the survey, as collecting new data will distort the state of the art before the pandemic. Moreover, people′s attitudes and perceptions about the quality and safety of vaccines manufacturing would be deeply influenced by the pandemic experience and by the rapid availability of COVID-19 vaccines. Therefore, making the same survey available again would result in a marked recall bias. For example, a survey involving the working-age population in France has highlighted that COVID-19 vaccine acceptance was lower for vaccines manufactured in China than for those manufactured in Europe [26]. However, a strong point of our study is that, to our knowledge, this is one of the first surveys investigating people’s perceptions and attitudes towards the vaccine production process. Enriching and deepening the knowledge about opinions and perceptions of the general population about vaccine manufacturing would surely be useful to improve the main topics to be communicated and to tackle vaccine hesitancy, providing correct information on vaccine manufacturing.

## 5. Conclusions

This paper aimed to enrich the knowledge about the perceptions and attitudes of the general population on vaccine manufacturing. This study shows that people seem to be confident in vaccine production. The main concern about vaccinations is related to a possible conflict of interest between companies and control systems. The next communication activities should also include some aspects of the vaccine production process. Lastly, it could be interesting to make the survey available online again in order to assess possible changes in people’s beliefs on the same issue after the availability of the new COVID-19 vaccines and their exceptionally rapid development and production.

## Figures and Tables

**Table 1:** Sociodemographic characteristics of respondents (*n* = 135).

Sociodemographic Characteristics of the Respondents.			
		*n*	% (*n*/N)
**Age group (years)**	20–29	48	35.6
	30–39	51	37.8
	40–49	18	13.3
	>49	18	13.3
**Sex**	Male	47	34.8
	Female	88	65.2
**Work or education in the healthcare setting**	Yes	47	34.8
	No	88	65.2
**Minor offspring**	Yes	38	28.1
	No	97	71.9

**Table 2:** Results of Section 2 of questionnaire: summary of the distribution of perceptions about vaccine production process stratified by sociodemographic characteristics. (Note—HC: Healthcare.).

Question	Response	Male (*n* = 47)		Female (*n* = 88)		Work/Education: HC (*n* = 47)		Work/Education: not HC (*n* = 88)		Minor Offspring (*n* = 38)		No Minor Offspring (*n* = 97)		Total (*n* = 135)	
		*n*	%	*n*	%	*n*	%	*n*	%	*n*	%	*n*	%	*n*	%
**I am concerned about** **the vaccine production**	Totally agree	2	4.26	5	5.68	1	2.13	6	6.82	3	7.89	4	4.12	7	5.19
	Partially agree	8	17.02	14	15.91	5	10.64	17	19.32	6	15.79	16	16.49	22	16.30
	Do not agree, do not disagree	4	8.51	7	7.95	5	10.64	6	6.82	4	10.53	7	7.22	11	8.15
	Partially disagree	4	8.51	14	15.91	7	14.89	11	12.50	5	13.16	13	13.40	18	13.33
	Totally disagree	29	61.70	48	54.55	29	61.70	48	54.55	20	52.63	57	58.76	77	57.04
	*p*-value	0.79				0.45				0.86					
**The vaccine vial does** **not contain harmful substances**	Totally agree	28	59.57	37	42.05	28	59.57	37	42.05	16	42.11	49	50.52	65	48.15
	Partially agree	6	12.77	31	35.23	8	17.02	29	32.95	11	28.95	26	26.80	37	27.41
	Do not agree, do not disagree	3	6.38	8	9.09	3	6.38	8	9.09	4	10.53	7	7.22	11	8.15
	Partially disagree	6	12.77	8	9.09	3	6.38	11	12.50	6	15.79	8	8.25	14	10.37
	Totally disagree	4	8.51	4	4.55	5	10.64	3	3.41	1	2.63	7	7.22	8	5.93
	*p*-value	0.06				0.06				0.51					
**During the vaccine production,** **the control system is adequate**	Totally agree	34	72.34	50	56.82	34	72.34	50	56.82	22	57.89	62	63.92	84	62.22
	Partially agree	7	14.89	27	30.68	9	19.15	25	28.41	7	18.42	27	27.84	34	25.19
	Do not agree, do not disagree	4	8.51	6	6.82	1	2.13	9	10.23	6	15.79	4	4.12	10	7.41
	Partially disagree	1	2.13	2	2.27	1	2.13	2	2.27	2	5.26	1	1.03	3	2.22
	Totally disagree	1	2.13	3	3.41	2	4.26	2	2.27	1	2.63	3	3.09	4	2.96
	*p*-value	0.34				0.25				0.08					
**There is a conflict of interest** **between manufacturing companies and control systems**	Totally agree	8	17.02	14	15.91	2	4.26	20	22.73	11	28.95	11	11.34	22	16.30
	Partially agree	15	31.91	29	32.95	12	25.53	32	36.36	13	34.21	31	31.96	44	32.59
	Do not agree, do not disagree	10	21.28	16	18.18	11	23.40	15	17.05	8	21.05	18	18.56	26	19.26
	Partially disagree	7	14.89	13	14.77	7	14.89	13	14.77	4	10.53	16	16.49	20	14.81
	Totally disagree	7	14.89	16	18.18	15	31.91	8	9.09	2	5.26	21	21.65	23	17.04
	*p*-value	0.98				<0.001				0.04					

**Table 3:** Results of Section 3 of questionnaire: summary of the distribution of perceptions about vaccine components stratified by sociodemographic characteristics. (Note—HC: Healthcare.).

Question	Response	Male (*n* = 47)		Female (*n* = 88)		Work/Education: HC (*n* = 47)		Work/Education: Not HC (*n* = 88)		Minor Offpsring (*n* = 38)		No Minor Offspring (*n* = 97)		Total (*n* = 135)	
		*n*	%	*n*	%	*n*	%	*n*	%	*n*	%	*n*	%	*n*	%
**Do you think that the microorganism from which the vaccine is derived is treated in a way that makes it harmless** **and unable to cause the disease?**	Yes	42	89.36	69	78.41	45	95.74	66	75.0	30	78.95	81	83.51	111	82.22
	No	5	10.64	19	21.59	2	4.26	22	25.0	8	21.05	16	16.49	24	17.78
	*p*-value	0.11				<0.001				0.53					
**Do you think that the amount of adjuvants (e.g., aluminum salts) in some vaccines is dangerous?**	Yes	4	8.51	14	15.91	6	12.76	12	13.64	7	18.42	11	11.34	18	13.33
	No	27	57.45	51	57.95	40	85.11	38	43.18	20	52.63	58	59.79	78	57.78
	I do not know what an adjuvant is	16	34.04	23	26.14	1	2.13	38	43.18	11	28.95	28	28.87	39	28.89
	*p*-value	0.38				<0.001				0.53					
**In your opinion, the precautionary withdrawal of some batches of vaccines indicates that the controls are:**	Ineffective and inadequate	5	10.64	17	19.3	2	4.26	20	22.73	5	13.16	17	17.53	22	16.30
	High effective so that suspect batches are immediately withdrawn	42	89.36	71	80.7	45	95.74	68	77.27	33	86.84	80	82.47	113	83.70
	*p*-value	0.19				0.01				0.54					

**Table 4:** Multivariate logistic regression analysis perfomed on 5-point Likert Scale statements (Section 2 of questionnaire). Note—β: regression coefficient. AOR: adjusted odds ratio.

Disagreement toward the Possibility of a Conflict Interest between Manufacturing Companies and the Control System					
Socio-Demographic Characteristic	β	AOR	SE	95% CI	*p*-Value
AGE (years)					
20–29	0.208	1.23	0.69	0.32–4.73	0.76
30–39	0.743	2.10	0.71	0.53–8.37	0.29
40–49	0.472	1.60	0.87	0.29–8.74	0.59
>49	Reference group	-	-	-	-
SEX					
Male	−0.046	0.95	0.43	0.41–2.2	0.91
Female	Reference group	-	-	-	-
HEALTHCARE WORK/EDUCATION					
Yes	0.889	2.43	0.41	1.08–5.48	0.03
No	Reference group		-	-	-
MINOR OFFSPRING					
Yes	−1.387	0.25	0.56	0.08–0.74	0.01
No	Reference group	-	-	-	-
Significance value H-L test = 0.462					

**Table 5:** Multivariate logistic regression analysis perfomed on Section 3 of questionnaire. Note—β: regression coefficient. AOR: adjusted odds ratio.

The Microorganism from Which the Vaccine Is Derived Is Treated in a Way That Makes It Harmless and Unable to Cause the Disease						
Socio-Demographic Characteristic	β	AOR	SE	95% CI		*p*-Value
AGE (years)						
20–29	−0.243	0.78	0.74	0.19–3.31		0.74
30–39	1.031	2.80	0.85	0.53–14.73		0.22
40–49	0.814	2.26	0.96	0.35–14.69		0.39
>49	Reference group	-	-	-		-
SEX						
Male	1.284	3.61	0.61	1.10–11.84		0.03
Female	Reference group	-	-	-		-
HEALTHCARE WORK/EDUCATION						
Yes	2.283	9.80	0.80	2.05–46.92		0.004
No	Reference group	-	-	-		-
MINOR OFFSPRING						
Yes	−0.802	0.45	0.65	0.13–1.59		0.21
No	Reference group	-	-	-		-
Significance value H-L test = 0.970						
**The Amount of Adjuvants in Some Vaccines Is Not Dangerous**						
	β	AOR	SE	95% CI		*p*-value
AGE (years)						
20–29	0.454	1.58	0.62	0.47–5.32		0.46
30–39	1.006	2.73	0.66	0.75–9.98		0.13
40–49	−0.361	0.70	0.78	0.15–3.2		0.64
>49	Reference group	-	-	-		-
SEX						
Male	1.284	1.64	0.44	0.69–3.91		0.26
Female	Reference group	-	-	-		-
HEALTHCARE WORK/EDUCATION						
Yes	1.923	6.84	0.49	2.63–17.82		<0.001
No	Reference group	-	-	-		-
MINOR OFFSPRING						
Yes	−0.154	0.86	0.50	0.32–2.31		0.32
No	Reference group	-	-	-		-
Significance value H-L test = 0.701						
**The Precautionary Withdrawal of Some Batches of Vaccines Indicates That the Controls Are High Effective**						
	β	AOR	SE	95% CI		*p*-value
AGE (years)						
20–29	−0.468	0.63	0.778	0.14–2.88		0.548
30–39	0.002	1.002	0.848	0.19–5.28		0.998
40–49	−0.217	0.80	0.977	0.12–5.46		0.824
>49	Reference group	-	-	-		-
SEX						
Male	0.922	2.51	0.588	0.79–7.96		0.117
Female	Reference group	-	-	-		-
HEALTHCARE WORK/EDUCATION						
Yes	2.126	8.38	0.789	1.79–39.31		0.007
No	Reference group	-	-	-		-
MINOR OFFSPRING						
Yes	0.397	1.49	0.657	0.41–5.39		0.545
No	Reference group	-	-	-		-
Significance value H-L test = 0.887						

## Data Availability

Data sharing is not applicable. Data were collected and managed in aggregated form according to European Union Regulation 2016/679 of the European Parliament and the Italian Legislative Decree 2018/101.

## Supplementary Materials

The following are available online at https://www.mdpi.com/article/10.3390/vaccines9091015/s1, File S1: Informed Consent, Questionnaire and Tables. Table S1: Results of Section 2 of questionnaire: summary of the distribution of perceptions about vaccine production process stratified by age groups. [NOTE. HC: Healthcare]; Table S2: Results of Section 3 of questionnaire: summary of the distribution of perceptions about vaccine components stratified by age groups.

## References

[1] World Health Organization (WHO) Immunization. https://www.who.int/news-room/facts-in-pictures/detail/immunization.

[2] WHO International Travel and Health Assessment. Chapter 6—Vaccine-Preventable Diseases and Vaccines (2019 Update). https://cdn.who.int/media/docs/default-source/travel-and-health/9789241580472-eng-chapter-6.pdf?.

[3] Dubé E., Laberge C., Guay M., Bramadat P., Roy R., Bettinger J.A. (2013). Vaccine hesitancy: An overview. Hum. Vaccines Immunother..

[4] Giambi C., Fabiani M., D’Ancona F., Ferrara L., Fiacchini D., Gallo T., Martinelli D., Pascucci M.G., Prato R., Filia A. (2018). Parental vaccine hesitancy in Italy—Results from a national survey. Vaccine.

[5] Bechini A., Bonanni P., Zanella B., Di Pisa G., Moscadelli A., Paoli S., Ancillotti L., Bonito B., Boccalini S. (2021). Vaccine Production Process: How Much Does the General Population Know about This Topic? A Web-Based Survey. Vaccines.

[6] Centers for Disease Control and Prevention (CDC) The Journey of Your Child’s Vaccine. https://www.cdc.gov/vaccines/parents/infographics/journey-of-child-vaccine.html.

[7] European Commission Good Manufacturing Practices (GMP) Guidelines. https://ec.europa.eu/health/documents/eudralex/vol-4_en.

[8] Preiss S., Garçon N., Cunningham A.L., Strugnell R., Friedland L.R. (2016). Vaccine provision: Delivering sustained & widespread use. Vaccine.

[9] WHO Regulation and Quality Control of Vaccines. https://www.who.int/biologicals/vaccines/regulation_and_quality_control_vaccines/en/.

[10] Tavakol M., Dennick R. (2011). Making sense of Cronbach’s alpha. Int. J. Med. Educ..

[11] Larson H.J., De Figueiredo A., Xiahong Z., Schulz W.S., Verger P., Johnston I., Cook A., Jones N.S. (2016). The State of Vaccine Confidence 2016: Global Insights Through a 67-Country Survey. EBioMedicine.

[12] Karafillakis E., Larson H.J. (2017). The benefit of the doubt or doubts over benefits? A systematic literature review of perceived risks of vaccines in European populations. Vaccine.

[13] Wilson R., Zaytseva A., Bocquier A., Nokri A., Fressard L., Chamboredon P., Carbonaro C., Bernardi S., Dubé E., Verger P. (2020). Vaccine hesitancy and self-vaccination behaviors among nurses in southeastern France. Vaccine.

[14] Williams S.E. (2014). What are the factors that contribute to parental vaccine-hesitancy and what can we do about it?. Hum. Vaccines Immunother..

[15] Paterson P., Meurice F., Stanberry L.R., Glismann S., Rosenthal S.L., Larson H.J. (2016). Vaccine hesitancy and healthcare providers. Vaccine.

[16] Yaqub O., Castle-Clarke S., Sevdalis N., Chataway J. (2014). Attitudes to vaccination: A critical review. Soc. Sci. Med..

[17] ECDC (2016). Let’s Talk about Hesitancy. https://www.ecdc.europa.eu/sites/portal/files/media/en/publications/Publications/lets-talk-about-hesitancy-vaccination-guide.pdf.

[18] Geoghegan S., O’Callaghan K.P., Offit P.A. (2020). Vaccine Safety: Myths and Misinformation. Front. Microbiol..

[19] Caudal H., Briend-Godet V., Caroff N., Moret L., Navas D., Huon J. (2020). Vaccine distrust: Investigation of the views and attitudes of parents in regard to vaccination of their children. Ann. Pharm. Françaises.

[20] WHO Vaccine Safety Advisory Committee (1999). Macrophagic myofasciitis and aluminum-containing vaccines. Wkly. Epidemiol. Rec..

[21] Baylor N.W., Egan W., Richman P. (2002). Aluminum salts in vaccines: US perspective. Vaccine.

[22] Descamps D., Hardt K., Spiessens B., Izurieta P., Verstraeten T., Breuer T., Dubin G. (2009). Safety of human papillomavirus (HPV)-16/18 AS04-adjuvanted vaccine for cervical cancer prevention: A pooled analysis of 11 clinical trials. Hum. Vaccines.

[23] Salk J.E., Bailey M.L., Laurent A.M. (1952). The use of adjuvants in studies on influenza immunization: II. increased antibody formation in human subjects inoculated with influenza virus vaccine in a water-in-oil emulsioN123. Am. J. Epidemiol..

[24] Boccalini S., Bonanni P., Chiesi F., Di Pisa G., Furlan F., Giammarco B., Zanella B., Tacconi F.M., Bechini A. (2020). The Experience of VaccinarSinToscana Website and the Role of New Media in Promoting Vaccination. Vaccines.

[25] Schmidt A.L., Zollo F., Scala A., Betsch C., Quattrociocchi W. (2018). Polarization of the vaccination debate on Facebook. Vaccine.

[26] Schwarzinger M., Watson V., Arwidson P., Alla F., Luchini S. (2021). COVID-19 vaccine hesitancy in a representative working-age population in France: A survey experiment based on vaccine characteristics. Lancet Public Health.

