# Supplementary material for: Quality and Safety of Vaccines Manufacturing: An Online Survey on Attitudes and Perceptions of Italian Internet Users
Source: Vaccines (Basel). 2021 Sep 13;9(9):1015. doi: 10.3390/vaccines9091015 (PMC8472959; doi:10.3390/vaccines9091015)
Supplement: Supplementary file 1 [file vaccines-09-01015-s001.zip › vaccines-1346441-supplementary.pdf]

vaccines

MDPI

Supplementary Materials

File S1: Informed Consent, Questionnaire and Tables

INFORMED CONSENT (BLANK FORM)

The aim of this survey is collecting information about general population's doubts concerning vaccine production process, in order to understand how this topic may influence the vaccine hesitancy.

The study is promoted by the Department of Health Sciences of the University of Florence.

The questionnaire is completely anonymous, and data will be collected and managed in aggregated form according to European Union Regulation 2016/679 of European Parliament and the Italian Legislative Decree 2018/101.

Do you agree to participate to the study?

[] I agree [] I do not agree

QUESTIONNAIRE

SECTION 1: SOCIODEMOGRAPHIC INFORMATION

- Age (years): select from 18 to 100

- Gender: [ ] M; [ ] F

- Do you work or have an education in the healthcare sector? [ ] Yes; [ ] No

- Do you have minor offspring? [ ] Yes; [ ] No

SECTION 2: PERCEPTIONS AND ATTITUDES TOWARD VACCINE PRODUCTION

How much do you agree with the following sentences?

- I am concerned about the vaccine production: [ ] Totally agree, [ ] Partially agree; [ ] Don't agree, don't disagree; [ ] Partially disagree; [ ] Totally disagree

- The vaccine vial does not contain harmful substances: [ ] Totally agree, [ ] Partially agree; [ ] Don't agree, don't disagree; [ ] Partially disagree; [ ] Totally disagree

- During the vaccine production, the control system is adequate: [ ] Totally agree, [ ] Partially agree; [ ] Don't agree, don't disagree; [ ] Partially disagree; [ ] Totally disagree

- There is a conflict of interests between manufacturing companies and the control systems: [ ] Totally agree, [ ] Partially agree; [ ] Don't agree, don't disagree; [ ] Partially disagree; [ ] Totally disagree

- Which are your main fears and concerns about vaccine production? (Open short answer)

SECTION 3: PERCEPTIONS AND ATTITUDES TOWARD VACCINE COMPONENTS

- Do you think the microorganism from which the vaccine is derived is treated in a way that makes it harmless and unable to cause the disease? [ ] Yes; [ ] No

- Do you think that the amount of adjuvants (e.g. aluminum salts) in some vaccines is dangerous? [ ] Yes; [ ] No; [ ] I do not know what an adjuvant is

- If you answered yes to the previous question, why? [ ] Adjuvants are harmful; [ ] The amount of adjuvant is excessive in some vaccines; [ ] Adjuvants are not adequately checked; [] Other

- In your opinion, the precautionary withdrawal of some vaccines batches reveals that the controls are: [ ] Ineffective and inadequate; [ ] High effective so that suspect batches are immediately withdrawn

SECTION 4: INFORMATION ABOUT VACCINES AND THEIR PRODUCTION

- Would you attend meetings regarding vaccines and their production? [ ] Yes; [ ] No; [ ] It depends on who organizes them

- Who should organize these events? (Multiple choice) [ ] Local agencies (e.g. municipalities, Region); [ ] Minister of Health; [ ] Medical Order; [ ] Educational Institute (e.g. schools, universities) [ ] Manufacturing companies; [ ] Other

Table S1. Results of Section 2 of questionnaire: summary of the distribution of perceptions about vaccine production process stratified by age groups. [NOTE. HC: Healthcare].

		20-29 years (N=48)		30-39 years (N=51)		40-49 years (N=18)		>49 years (N=16)		Total (N=135)		*p*-value
		**n**	**%**	**n**	**%**	**n**	**%**	**n**	**%**	**n**	**%**	
**I am concerned about the vaccine production**	Totally agree	2	4.17	3	5.88	1	5.56	1	5.56	7	5.19	0.30
	Partially agree	6	12.50	7	13.73	6	33.33	3	16.67	22	16.30	
	Don't agree, don't disagree	3	6.25	5	9.80	0	0.00	3	16.67	11	8.15	
	Partially disagree	5	10.42	7	13.73	1	5.56	5	27.78	18	13.33	
	Totally disagree	32	66.67	29	56.86	10	55.56	6	33.33	77	57.04	
**The vial does not contain harmful substances**	Totally agree	22	45.83	24	47.06	8	44.44	10	55.56	64	47.41	0.78
	Partially agree	14	29.17	12	23.53	8	44.44	4	22.22	38	28.15	
	Don't agree, don't disagree	5	10.42	4	7.84	1	5.56	1	5.56	11	8.15	
	Partially disagree	4	8.33	6	11.76	1	5.56	3	16.67	14	10.37	
	Totally disagree	3	6.25	5	9.80	0	0.00	0	0.00	8	5.93	
**During the vaccine production, the control system is adequate**	Totally agree	29	60.42	31	60.78	14	77.78	9	50.00	83	61.48	0.23
	Partially agree	17	35.42	11	21.57	1	5.56	6	33.33	35	25.93	
	Don't agree, don't disagree	1	2.08	5	9.80	3	16.67	1	5.56	10	7.41	
	Partially disagree	0	0.00	2	3.92	0	0.00	1	5.56	3	2.22	
	Totally disagree	1	2.08	2	3.92	0	0.00	1	5.56	4	2.96	
**There is a conflict of interest between manufacturing companies and the control system**	Totally agree	6	12.50	7	13.73	6	33.33	3	16.67	22	16.30	0.19
	Partially agree	12	25.00	20	39.22	4	22.22	8	44.44	44	32.59	
	Don't agree, don't disagree	13	27.08	6	11.76	4	22.22	3	16.67	26	19.26	
	Partially disagree	8	16.67	6	11.76	2	11.11	4	22.22	20	14.81	
	Totally disagree	9	18.75	12	23.53	2	11.11	0	0.00	23	17.04	

Table S2. Results of Section 3 of questionnaire: summary of the distribution of perceptions about vaccine components stratified by age groups.

		**20-29 years (N=48)**		**30-39 years (N=51)**		**40-49 years (N=18)**		**>49 years (N=16)**		**Total (N=135)**		***p*-value**
		**n**	**%**	**n**	**%**	**n**	**%**	**n**	**%**	**n**	**%**	
**Do you think that the microorganism from which the vaccine is derived is treated in a way that makes it harmless and unable to cause the disease?**	Yes	38	79.2	44	86.3	15	83.3	14	77.8	111	82.2	0.76
	No	10	20.8	7	13.7	3	16.7	4	22.2	24	17.8	
**Do you think that the amount of adjuvants (e.g. aluminum salts) in some vaccines is dangerous?**	Yes	4	8.3	5	9.8	5	27.8	4	22.2	18	13.3	0.07
	No	30	62.5	35	68.6	6	33.3	7	38.9	78	57.8	
	I dont' know what an adjuvant is	14	29.2	11	21.6	7	38.9	7	38.9	39	28.9	
**In your opinion, the precautionary withdrawal of some batches of vaccines indicates that the controls are:**	Ineffective and inadequate	9	18.8	7	13.7	3	16.7	3	16.7	22	16.3	0.92
	So effective that suspect batches are immediately withdrawn	39	81.3	44	86.3	15	83.3	15	83.3	113	83.7	
